# Profiling of Mitochondrial DNA Heteroplasmy in a Prospective Oral Squamous Cell Carcinoma Study

**DOI:** 10.3390/cancers12071933

**Published:** 2020-07-17

**Authors:** Liane Fendt, Federica Fazzini, Hansi Weissensteiner, Emanuel Bruckmoser, Sebastian Schönherr, Georg Schäfer, Jamie Lee Losso, Gertraud A. Streiter, Claudia Lamina, Michael Rasse, Helmut Klocker, Barbara Kofler, Anita Kloss-Brandstätter, Christian W. Huck, Florian Kronenberg, Johannes Laimer

**Affiliations:** 1 Institute of Genetic Epidemiology, Department of Genetics and Pharmacology, Medical University of Innsbruck, A-6020 Innsbruck, Austria; liane.fendt@i-med.ac.at (L.F.); federica.fazzini@i-med.ac.at (F.F.); Hansi.Weissensteiner@i-med.ac.at (H.W.); sebastian.schoenherr@i-med.ac.at (S.S.); jamie_lee1515@hotmail.com (J.L.L.); gertraud.erhart@i-med.ac.at (G.A.S.); claudia.lamina@i-med.ac.at (C.L.); A.Kloss-Brandstaetter@fh-kaernten.at (A.K.-B.); Florian.Kronenberg@i-med.ac.at (F.K.); 2 Oral and Maxillofacial Surgeon, Private Practice, A-5020 Salzburg, Austria; research@bruckmoser.info; 3 Institute for Pathology, Neuropathology and Molecular Pathology, Medical University of Innsbruck, A-6020 Innsbruck, Austria; georg.schaefer@i-med.ac.at; 4 University Hospital for Craniomaxillofacial and Oral Surgery, Medical University of Innsbruck, A-6020 Innsbruck, Austria; michael.rasse@tmo.at; 5 Clinic for Maxillofacial Surgery, Sechenov University, Trubetskaya Str. 8 b.2, 119992 Moscow, Russia; 6 Division of Experimental Urology, Department of Urology, Medical University of Innsbruck, A-6020 Innsbruck, Austria; helmut.klocker@i-med.ac.at; 7 Department of Otorhinolaryngology, Medical University of Innsbruck, Anichstrasse 35, A-6020 Innsbruck, Austria; ba.kofler@tirol-kliniken.at; 8 Carinthia University of Applied Sciences, A-9524 Villach, Austria; 9 Institute of Analytical Chemistry and Radiochemistry, CCB-Center for Chemistry and Biomedicine, Leopold Franzens University Innsbruck, A-6020 Innsbruck, Austria; Christian.W.Huck@uibk.ac.at

**Keywords:** oral cancer, oral squamous cell carcinoma, OSCC, heteroplasmy, mitochondrial DNA, mtDNA, next generation sequencing, NGS, survival analysis, haplogroup

## Abstract

While a shift in energy metabolism is essential to cancers, the knowledge about the involvement of the mitochondrial genome in tumorigenesis and progression in oral squamous cell carcinoma (OSCC) is still very limited. In this study, we evaluated 37 OSCC tumors and the corresponding benign mucosa tissue pairs by deep sequencing of the complete mitochondrial DNA (mtDNA). After extensive quality control, we identified 287 variants, 137 in tumor and 150 in benign samples exceeding the 1% threshold. Variant heteroplasmy levels were significantly increased in cancer compared to benign tissues (*p* = 0.0002). Furthermore, pairwise high heteroplasmy frequency difference variants (∆HF% > 20) with potential functional impact were increased in the cancer tissues (*p* = 0.024). Fourteen mutations were identified in the protein-coding region, out of which thirteen were detected in cancer and only one in benign tissue. After eight years of follow-up, the risk of mortality was higher for patients who harbored at least one ∆HF% > 20 variant in mtDNA protein-coding regions relative to those with no mutations (HR = 4.6, (95%CI = 1.3–17); *p* = 0.019 in primary tumor carriers). Haplogroup affiliation showed an impact on survival time, which however needs confirmation in a larger study. In conclusion, we observed a significantly higher accumulation of somatic mutations in the cancer tissues associated with a worse prognosis.

## 1. Introduction

The oral squamous cell carcinoma (OSCC) represents over 90% of the most frequent types of cancer in the oral cavity and accounts for 38% of head and neck tumors [1]. Tumor incidence has increased up to approximately 300,000 newly diagnosed cases per year and 2.1% of total cancer deaths worldwide. Known risk factors include tobacco use in any form, especially in combination with heavy alcohol consumption [2]. Unfavorably, the prognosis of OSCC is generally poor. The five-year survival rate of OSCC is only 50% and has remained unchanged for a decade [3].

Mitochondria have been implicated in carcinogenesis due to their crucial role in energy metabolism. The mitochondrial genome comprises 37 distinct genes of which 13 encode for oxidative phosphorylation (OXPHOS) proteins involved in the electron transport chain of the aerobic energy production of the cell. The mutation rate within the mitochondrial DNA (mtDNA) is known to be up to 10 times higher compared to the nuclear DNA [4,5].

MtDNA mutations emerge as heteroplasmies, where a first hit in one out of hundreds of mtDNA molecules within a cell may reach any level of mixture between wild-type and variant. If such a mutation substitutes all wildtype bases, the mutations are referred to as homoplasmies, variants, or polymorphisms. The majority of those fixed exchanges are neutral. Constantly emerging distinct patterns of polymorphisms (haplogroups) in the course of the evolution are used to reconstruct the human phylogeny [6,7,8]. Presumable associations of these polymorphisms to cancer risk have led to on-going discussions [9].

In contrast, the presence of heteroplasmy affecting functionally relevant sites might be an indication for disease and is found in many human tumors [10]. In fact, our group could demonstrate the involvement of mtDNA mutations in several types of cancer [11,12,13,14]. Several studies reported a potential role of mtDNA mutations in OSCC [15,16,17,18,19,20,21,22]. The majority, however, suffered from limitations such as low sensitivity Sanger sequencing on a very small part of the mtDNA genome, like the D-Loop that undermined the original claims. Only a few studies presented data from deep next-generation sequencing of the whole mtDNA [18,23,24]. In addition to this, the literature displays sequence artifacts [25] misinterpreted as pathogenic mtDNA mutation emphasizing the necessity for the establishment of a reliable sequencing strategy. Recently, we started to investigate the mutational context in OSCC based on an emerging sequencing technology, which we then validated by gold-standard Sanger sequencing [14]. Preliminary data from this report showed point mutations in the tumor fractions. Therefore, we elaborated a sophisticated next-generation sequencing (NGS) pipeline for the laboratory and the data analysis in order to assess low-level heteroplasmies with high accuracy [26,27]. For data analysis, mutserve (https://github.com/seppinho/mutserve) and haplocheck (https://github.com/genepi/haplocheck [28]) have been used. Applying this new pipeline, we investigated in the present study low-level mtDNA point heteroplasmy in 37 paired specimens (37 OSCC and 37 matched benign tissues). Furthermore, the availability of prospective data with a median follow-up of eight years allowed us to evaluate the association of mitochondrial mutations and haplogroup background with mortality risk.

## 2. Results

### 2.1. Coverage and Haplogroup Classification 

The plausibility of the resulting sequences was checked by the reconstruction of the phylogenetic relatedness between the individual haplotypes. Haplogroups were assigned with HaploGrep2 according to the nomenclature in Phylotree 17 [29,30]. As expected, the mtDNA sequence patterns of the benign and the matching cancer samples of each patient were successfully assigned to identical haplogroups. All investigated sequences belonged to typical European mitochondrial lineages [6]. The majority of the sequences belonged to haplogroup H (22 out of 37, 59.5%), while the remaining samples were classified into the haplogroup UK-cluster (9, 24.3%), JT-cluster (5, 13.5%) and W (1, 2.7%). Table 1 displays patient characteristics and haplogroups.

**Figure 1:**
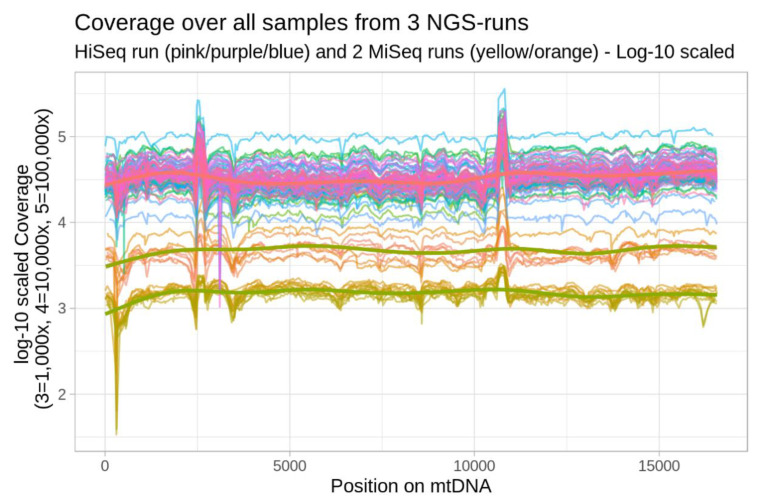
Distribution of the per-base coverage for all 74 specimens sequenced in the three different runs. The X-axis represents the mitochondrial positions 1–16,569, while the Y-axis displays the perbase read coverage on a log−10 scaled axis. The mean coverage depth was 5210× for MiSeq run one, 1547× for the MiSeq run two and 36,220× for the previously published HiSeq data.

The sequence coverage of the 74 specimens sequenced in three different runs (two runs on Illumina MiSeq and one run on Illumina HiSeq) is shown in Figure 1. The median and mean coverage depth were 4730× and 5210× respectively for MiSeq run one (*n* of samples = 10), 1516× and 1547× respectively for the second MiSeq run (*n* = 8), and 32,505× and 36,220×, respectively, for the previously published HiSeq data (*n* = 56) [14]. The highest concordance could be observed between coverage depth in the HiSeq samples (R = 0.96), with MiSeq Run 1 and 2 showing a lower homogeneity with R = 0.85 and R = 0.92, respectively. The lowest correlation was seen between HiSeq and MiSeq run two (R = 0.72).

The representation in Figure 1 indicates the deviation from the expected homogenous read distribution over the whole mitochondrial genomes based on the equimolar DNA library pool. Two regions of over-represented coverage are visible between positions 2480 and 2688, as well as between positions 10,653 and 10,858 reflecting the regions where primers overlap, which causes a relative double amount of DNA library fragments. The mitochondrial homopolymeric stretch around 309 relative to the rCRS is comparatively underrepresented due to quality cutoff settings for multiple consecutive alignments.

### 2.2. Quality Control, NUMTS and Contamination Detection

Two of the sample pairs (KT012, KT013) exceeded the average heteroplasmy count of the other samples by more than three-fold. A detailed inspection of the heteroplasmic sites showed the presence of apparent NUMTs polymorphisms (mtDNA sequences transferred into nuclear DNA) within these two sample pairs evidenced by a distinct mutational pattern at positions 2523, 2541, 2542, 2543, 2557, 2560, 2563, 2567, 2570, 2572, 2577, 2581, 2589, 2600, 2625, 2628, 2640, 2645, 2647, 2667, 2683, 2686, 2687, 2702 and 2747 at homogenous levels of ~1.7% in KT012 and up to 8.6% in KT013. The co-amplification of NUMTs is further supported by previous data showing the same low-level heteroplasmic pattern within three samples (MKG1 cancer, MKG5 benign and cancer), previously attributed to PCR primer annealing issues [14]. Consequently, the sensitivity of the NGS sequence variant calling by our analysis pipeline enables the identification of low-level heteroplasmy. It further shows that a critical assessment of the results is fundamental to encompass the detection of low-level contamination and potential NUMTs. Recent studies have revealed that mega-NUMTs can resemble complete haplotypes [31,32,33]. Sample contamination checks using Haplocheck lead to the exclusion of one sample pair (MKG16), so that 36 sample-pairs were analyzed in the subsequent steps.

### 2.3. Mitochondrial DNA Low-Level Heteroplasmic Point Variants in Oral Cancer Sample Pairs

**Figure 2:**
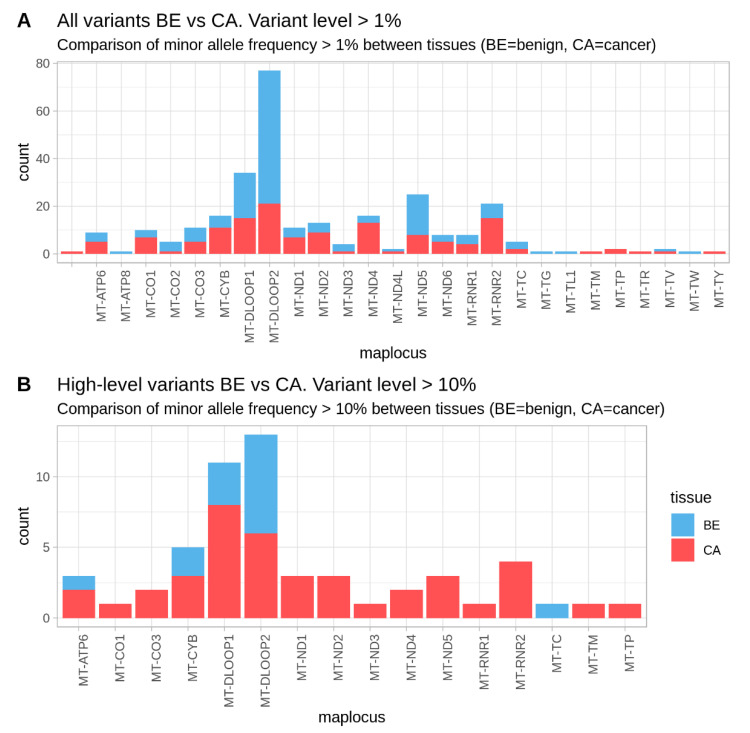
Number of somatic variants in the different regions of the mitochondrial genome in benign (blue) and cancer (red) tissues. Comparison between the number of variants including all heteroplasmic variants above 1% (**A**) and above 10% minor allele frequency (**B**). Low-level heteroplasmies were more frequent in benign samples and they were mostly localized in the control region (D-Loop 1 and 2) (A). On the contrary, the majority of high-level heteroplasmies have been observed in the cancer samples and within coding regions (B).

The accumulation of heteroplasmic sites was different between distinct mitochondrial regions, as well as between benign and tumors specimens. The total number of heteroplasmies above 1% was 287 (150 variants in benign tissues, 137 in cancer tissues). In the benign tissues, exactly half of these variants (75 out of 150) were observed in the control region (D-loop 1 and D-Loop 2), while in cancer tissue they represented only 26.3% (36 out of 137) (Figure 2A). The analysis of the tumor specimens showed that while the non-coding control region (CR) harbored most heteroplasmic sites to the base-pair ratio (9.89% of all bases within CR), the mitochondrial OXPHOS genes and tRNAs, as well as rRNA genes incorporated less (1.15%, 0.99% and 1.15% of all bases within the coding regions). When applying a 2% threshold the number decreased to 146 (62 heteroplasmic sites in benign and 84 in cancer tissue), which showed that almost half (45.6%) of the variants detected had a heteroplasmic level below 2%. A different picture was observed for high-level heteroplasmic variants (>10%). Forty out of 54 (74%) variants were found in cancer specimens and two-thirds (67.5%) of them localized in the coding regions. In contrast, benign tissue samples harbored only 14 (26%) high-level heteroplasmic variants with four (28.6%) of them localized in the coding regions (Figure 2B).

**Figure 3:**
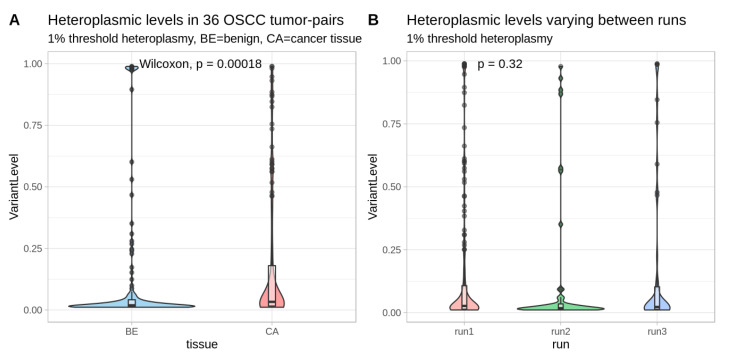
Violin- and Boxplots showing heteroplasmy levels in 36 sample pairs. The median heteroplasmy levels in the benign tissues corresponding to 1.9% are lower than in cancer tissues with a median of 3.3% when applying a 1% threshold. The *p*-value was derived from the Wilcoxon test (**A**). Heteroplasmic variant levels in the three different sequencing runs (run1: HiSeq run filtered with *n* = 27, run2: Miseq run with *n* = 5, run3: MiSeq run with *n* = 4) with the cutoff at 1%. The *p*-value was derived from the Kruskal–Wallis test (**B**).

The median level of observed heteroplasmic variants in the coding regions was significantly higher in the cancer samples (median 3.3%, vs. 1.9% *p*-value = 0.0002, Figure 3A). We did also note a difference in the variant levels per run, however not statistically significant, and is represented in Figure 3B filtered for 1% heteroplasmic levels.

The frequency table of the variants found in cancer, as well as benign tissues, shows mostly singletons, or variants specific to a benign and tumor sample pair (72.6% of all variants, Table S1). As expected, most heteroplasmic variants which could be found in more than one sample were present in the D-Loop 1 and 2 (14 sites), with only one site in rRNA (709), and two sites in coding genes (3210 and 14560). Interestingly, position 72 was the site with the highest frequency being present 15 times (12 times in benign samples and three times in cancer samples) and exclusively found in haplogroup H. Using the 1000G phase 3 data as the control group, which we also previously analyzed with mtDNA-Server and haplocheck [26,28], only four samples out of 2504 had a heteroplasmic variant on position 72. All four samples were also exclusive to the haplogroup HV-cluster. The situation was similar with the transversion on 414 T > G found in eight benign and four OSCC samples, which could only be detected in one sample of the complete 1000 G data set.

### 2.4. Mutations with High Heteroplasmy Difference Between Cancer and Benign and Their Clinical Impact

**Figure 4:**
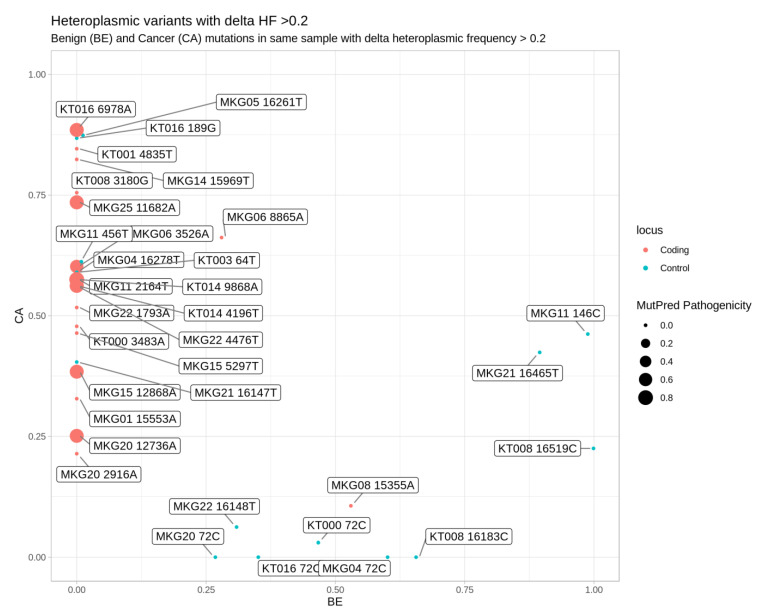
Mitochondrial variations with delta heteroplasmic frequency between benign and cancer tissue of at least 20%. In the graph are displayed: on the axes, the variant frequency in the cancer tissue (y-axis) and the variant frequency in the benign tissue (x-axis), in rectangle labels the genome positions and sample ID, with the MutPred Pathogenicity Score represented by the point size and colored according to the presence in either coding region including rRNA and tRNA variants or control region (D-Loop 1 and D-Loop 2).

To reduce the number of possible artifacts when analyzing the clinical impact of variants, which also need to exceed higher thresholds to be functionally relevant [34], we analyzed the data using the approach described by Hopkins et al. [35]. In total, 34 mtDNA variants with a heteroplasmy level difference between tumor and paired benign samples of at least 20% (∆HF% > 20%) were identified. Figure 4 and Table S2 show these variants, 24 of which displayed a higher heteroplasmy level in cancer than in benign tissue (24 out of 34 = 70.6%, Fisher’s exact test *p* = 0.0014). Fourteen of these mutations (all transitions) were located in the coding region and eight lead to amino acid substitutions. To predict a functional impact a quantitative predictive score approach combining phylogenetics and pathogenicity scoring [36] was applied. Four of the detected high-level heteroplasmies (9868R, 4196Y, 6978R, 11682R) in mitochondrial genes were characterized as high pathogenicity score (≥0.7) mutations. This assignment was further supported by the fact that none of them were known from the phylogeny. In contrast, only one (MKG5, 15355A, synonymous mutation) out of 14 ∆HF% > 20 mtDNA variants located in the coding region, had a higher heteroplasmy level in benign tissue (Fisher’s exact test *p* = 0.0240).

**Figure 5:**
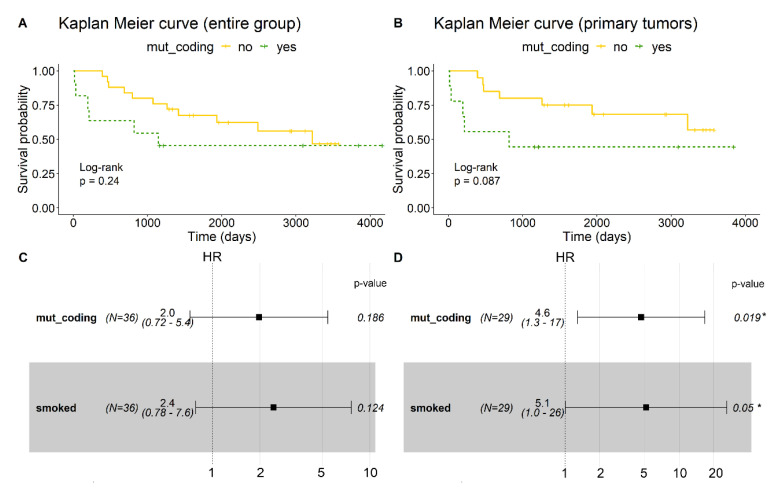
Kaplan–Meier curve for the entire patient group, *n* = 36 (**A**) and only in patients with primary tumors, *n* = 29 (**B**). Patients who harbored at least one mutation in the mtDNA protein-coding region (green dashed line) had poorer survival rates than patients who did not harbor mutations (yellow straight line). Forest plots of cox proportional-hazard models for mut_coding adjusted for smoking status are shown as: (**C**) for the entire group (HR = 2.0 (95%CI = 0.72–5.4), *p* = 0.186) and (**D**) for primary tumors (HR = 4.6 (95%CI = 1.3–17), *p* = 0.019). Mut_coding = ∆HF% > 20 mutations in mtDNA protein-coding region. *p-value ≤0.05

We performed a survival analysis considering only these 34 potentially deleterious variants with high heteroplasmy frequency difference (∆HF% > 20). A total of 20 deaths occurred during a median follow-up of eight years (IQR: (1961 days; 3472 days)). The characteristics of the patients are presented in Table 1. The cause of death was cancer for 17 patients (85% of all the deaths). Kaplan–Meier curves indicated that survival was shorter in patients whose tumors harbored at least one ∆HF% > 20 mutation in mtDNA protein-coding regions (mut_coding) relative to those with no mutations (Figure 5A). The median survival time was 1144 days (38 months) for the patients carrying mut_coding variants and 3222 days (107 months) for patients without mtDNA mutations, although this result did not reach statistical significance (*p* = 0.24). The effect was more pronounced when we only analyzed patients with primary tumors (*n* = 29). Figure 5B shows an increasing trend in mortality risk for primary tumor patients when mut_coding variants were present (*p* = 0.087). Univariate Cox proportional-hazard models for mut_coding and other potential risk factors did not show any significant associations (Table S3). Explorative analyses showed, however, that there were fewer smokers among mut_coding carriers (7 out of 11 = 63.6%) than among non-mutation carriers (17 out of 25 = 68%). Since smoking is a known strong risk factor for oral cancer [37], the higher rate in non-mutation carriers might mask the true risk. Therefore, we adjusted for smoking status, leading to an HR of 2.0 (*p* = 0.186, Figure 5C) for all and of 4.6 for primary tumor carriers (*p* = 0.019, Figure 5D). Considering the small number of events, performing a Cox regression model with more variables is not feasible and therefore, we consider this model, adjusted only for smoking status, as the main model. As a sensitivity analysis, we additionally adjusted for the presence of regional lymph node metastases (N) to exclude potential confounding, since its univariate HR was in the same ballpark as for mut-coding and smoking status. However, the HR barely changed (HR = 2.1, *p* = 0.157 additionally adjusted for N).

Interestingly, the haplogroup distribution in the patients was different from other published studies from the same (or nearby) geographic region. In particular, the frequency of haplogroup H was greater in OSCC patients compared to the general population from the Salzburg Saphir study [38] (*n* = 1598) and the Austrian juvenile obese cohort in Styria—STYJOBS/EDECTA (*n* = 251). In our study, 59.5% of the patients belong to haplogroup H, while this haplogroup represented 44% in the Saphir study (*p* = 0.06), 42% in the STYJOBS/EDECTA (*p* = 0.044) and 38% in patients recruited in the exact same hospital, who underwent radical prostatectomy (*n* = 50, *p* = 0.047) [13]. Furthermore, in our study, the risk of cancer mortality was higher for haplogroup H patients relative to those of other haplogroups (HR = 2.1 (95%CI = 0.73–6.10); *p* = 0.165 adjusted for smoking, Figure S1). Furthermore, individuals within the UK haplogroup cluster (*n* = 8) were significantly older with a median age of 71.5 years whereas those in Haplogroup H (*n* = 22) showed a median age of 59.5 years (*p* = 0.031, Figures S2 and S3).

## 3. Discussion

The unambiguous identification of potentially pathological mtDNA mutations is crucial for the interpretation and functional assessment of any aspects of tumorigenesis. In this study, we presented an extensive profiling of the mitochondrial genome in OSCC. We identified a heterogeneous distribution of heteroplasmic sites in the different mitochondrial regions. The trend in our data is in agreement with data from the general population (1000 Genomes project) [39] and reflects the lacking selection pressure on non-functional parts of the mitochondrial control region, while mutations in the gene coding regions are functionally sensitive towards mutational loads. We further identified a tendency of accumulating high-level heteroplasmies with the potential for functional impact exclusively in cancer tissues. This is in line with the results of a recent study of OSCC in the Indian population [23] but it was also reported for other cancer types [11,12,40]. Prospective studies that examine the association between mtDNA mutations and clinical outcomes in OSCC patients are scarce [22,23]. Despite the limited sample size, in our study we found that mutations in the coding regions and in haplogroup H were associated with worse outcomes.

However, not only the level of heteroplasmy but also the absolute mitochondrial capacities reflected by the remaining number of intact wild-type mtDNA molecules define the potential to change the cell’s metabolism based on these mutations. This issue remains for further investigation.

Tumor grading, T-stage [41], and perineural infiltration [42] are major prognostic factors in OSCC patients. Furthermore, the formation of local metastases into loco-regional lymph nodes plays a prognostically significant role. Factors negatively influencing survival time include the number of lymph nodes affected, the presence of extranodular spread, as well as the involvement of caudal lymph node levels (V + IV) [41,43,44,45,46]. Patients with a tumor of grade 3 showed a significantly lower age (median 54.5 years) compared to grade 2 (median 62 years) *p* = 0.033 (Figure S4). However, Cox-regression analysis for grading alone, as well as adjustment for smoking and age did not reach statistical significance.

The search for serum tumor markers for OSCC was not successful so far, there is still no application for the clinical routine [47,48,49]. In this regard, it is conceivable that mutations in the mtDNA, which is abundant at 100- to 1000-fold compared to nuclear DNA, could be detected in a blood sample and serve as a potential diagnostic marker. This idea has to be investigated in future studies.

Only a few studies used next-generation sequencing techniques and investigated heteroplasmic variants in the whole mtDNA [18,23,24]. Recently, Palodhi et al. [23] investigated tumor tissue and matched blood samples from 89 OSCC patients. The authors showed that patients with mtDNA non-synonymous somatic mutations had a higher probability of suffering from lymph node metastasis than those without. Moreover, the ratio of non-synonymous to synonymous mutations in cancer tissue was higher than in matched blood samples, showing a positive selection of non-synonymous mutations in OSCC tumors, similar to what we also observed in our samples. Surprisingly, another study recently published [24] detected 166 somatic point mutations in half of the cancer samples (14 out of 28) and none in the other half of the samples. However, in line with our results, the majority of the identified mutations were located in the protein-coding region.

In another study, it was demonstrated that some patients with recurrent head and neck squamous cell carcinoma (HNSCC) but not those without recurrences had mtDNA mutations in their histologically negative margins, suggesting the potential usefulness for disease monitoring [50]. Therefore, we hypothesize that patients who are histologically classified as tumor-free but have marginal cut-outs might benefit from shorter follow-up intervals to confirm a postulated molecular in sano state. Further support for this idea came from Kumar et al. [51] evidencing that the presence of cfmtDNA (cell-free mtDNA) in liquid biopsies of patients with head and neck squamous cell carcinoma could be used as an early diagnostic marker. Recent research efforts investigated mtDNA as an outcome predictor for other tumors. Cicchillitti et al. [52] quantified cell-free DNA, as well as the relative mitochondrial cell-free DNA by qPCR in the endometrium. Their data indicate that assessment of total and mitochondrial cell-free DNA levels in blood sera and the relative NLR and MLR (neutrophil-to-lymphocyte (NLR) and monocyte-to-lymphocyte (MLR) ratios) in blood obtained from patients preoperatively may be helpful for the clinical management and improve prognostic predictions in endometrial cancer. Investigations on prostate cancer by Creed et al. [53] further indicated the possibility of detecting changes in the mtDNA by a simple blood test. This could reduce the re-biopsy rate since a particular mtDNA deletion was shown to be a strong predictor for clinically significant prostate cancer independent of PSA or age. It can be speculated that in OSCC patients, saliva rather than a blood test could be used for cancer-specific early prognostic marker detection.

Taking into account our results, as well as those of the above-mentioned studies, detection of mtDNA mutations in OSCC follow-up could potentially provide advantages over other existing methods. One of these potential advantages is the obvious higher sensitivity for tumor-specific variant detection, especially compared to existing cytologic techniques based on microscopic detection of tumor cells. Our data in particular evidence the sensitivity for detecting mtDNA variants down to a 1% level minor allele contribution. The development of our bioinformatics pipeline reflects an automated analysis tool for mtDNA NGS data, including a check for contamination and sample mix up with Haplocheck (v1.0.11 paper in preparation [28]).

Another potential advantage of an mtDNA based follow-up is that the surgically resected tumor tissue or biopsy sample generally provides relatively pure tumor DNA. This allows for easy identification of patients who may be suitable for an mtDNA-based follow-up [54]. Furthermore, the rapid development of emerging sequencing technologies including sophisticated bioinformatics solutions for big data handling is bound to facilitate the assessment of target regions. Continuously decreasing analysis time frames and costs can be expected, which is amazingly demonstrated by continuous developments by the Oxford Nanopore systems [55].

Finally, OSCC represents a tumor entity with the early release of tumor cells into the saliva. If specific mutations are present, it is likely that these mtDNA changes can be detected with higher sensitivity compared to cytologic identification of tumor cells.

### Strenghts and Limitations

The major strength includes the availability of outcome data with a long follow-up period. Furthermore, the comparison of tumor and corresponding benign tissue is especially important because this approach is best suited to identify the presence of tissue-specific differences at the heteroplasmy level. Often tumor samples are compared to blood samples, although it is known that accumulation of mtDNA variants is tissue-specific, even within individuals [56,57]. Analyzing variants with a difference of the heteroplasmy level percent >20 renders our analysis stable against potentially shared variants in the control tissue.

This study also has some limitations. This investigation includes the deep sequencing of the entire mitochondrial genome, with an average coverage of ~26,000×, however, differing considerably between the runs, see Figure 1. This coverage difference did not affect the results as the heteroplasmic variant levels were not statistically different (Figure 3). The median coverage for four pairs (eight samples) was only 1516×. However, in the survival analysis, we focused on higher-level mutations (∆HF% > 20) for which the coverage is not so crucial. The OSCC tissue samples were not homogenous: seven specimens were collected from recurrent cancers, while the rest were taken from primary tumors. This could affect the mutation profile. Additionally, we were limited to compare cancer tissues to “normal” tissues, i.e., benign oral squamous cells, as neither blood samples nor saliva were available for this study. This is especially interesting since smoking exposes normal mucosa cells to carcinogens. We found a similar pattern of mutations in coding regions between smokers and non-smokers. However, the question remains about how the carcinogens of cigarette smoking could affect other tissues as well. A further limitation of our study is that for some analysis the sample size was too small to reach significance levels. Especially, in the Cox regression analysis the limited number of outcomes did not allow us to consider all potential confounders. However, we investigated all univariate Cox-models of available phenotypes (see Table S3) and based our selection on those with high hazard-ratios as well.

## 4. Materials and Methods

### 4.1. Samples

The inclusion criteria for the study were as follows: age over 18 years, histologically proven diagnosis of OSCC and written informed consent for study enrollment. Exclusion criteria comprised punch biopsies containing less than 70% of tumor-specific cells.

Tumor and corresponding benign mucosa cells of 37 individuals were collected from 2 mm biopsies directly upon surgery. Normal mucosa cells were taken from the contralateral side of the oral cavity. Samples were immediately frozen at –80 °C and transferred to the Institute of Pathology for the diagnostic work-up. Diagnosis was performed on several cryo-sections, and tumor status and tumor cell content of the biopsies were verified. Samples were then stored frozen until DNA extraction.

The study was approved by the Ethical Committee of the Medical University of Innsbruck (Ref. No. AN2016-0026 359/4.2 366/5.8 (3923a)) and has been carried out in accordance with the Code of Ethics of the World Medical Association (Declaration of Helsinki). All patients participated in the study upon written informed consent. The privacy rights of all subjects have been observed throughout.

### 4.2. Clinical Endpoint and Follow-up

The routine clinical and radiological follow-up comprised an appointment in our specialized tumor clinic every three months during the first two years with both a clinical examination and a radiological control by sonography or computed tomography. Following this period, intervals were extended to half a year (i.e., two times per year) for an additional three-year period including a clinical exam and a computed tomography. Finally, patients returned once a year for a clinical exam and a computed tomography from the sixth to tenth year (referring to the completion of initial therapy).

### 4.3. Extraction and Amplification of Mitochondrial DNA

Total genomic DNA was isolated with the QIAGEN EZ1 DNA extraction robot (Qiagen, Hilden, Germany) and quantified by TECAN infinite M200 Nanoquant (Tecan Group Ltd., Männedorf, Switzerland). Whole mitochondrial genomes were amplified as 2 × 8.5 kb fragments (A, B) using primers described by Fendt et al. [58]. Long-distance amplification was performed using NEB Long Amp Taq polymerase (New England Biolabs, Ipswich, MA, USA) according to the manufacturer’s protocol.

### 4.4. Next-Generation Sequencing of Mitochondrial Genomes

Libraries were constructed starting from 200 ng of pooled PCR fragments A and B of each sample. In a first step, the pooled samples were enzymatically fragmented to 500–800 basepair in a final volume of 20 µL, containing 2 µL of 2× dsDNA Fragmentase Reaction Buffer v2 and 2 µL of 200 mM MgCl2 and 2 µL NEBNext dsDNA Fragmentase (New England Biolabs, Ipswich, MA, USA) by incubation for approximately 6 min at 37 °C. The resulting fragments were purified using MagSi-NGSPREP beads (MagnaMedics Diagnostics, Geleen, Netherlands). Dual-indexed libraries were produced using the TruSeq Nano DNA HT Sample Preparation Kit (llumina, Inc., San Diego, CA, USA) according to the manufacturer’s instruction with minor modifications: for right side size selection, 47.5 µL and for left side size selection, 15 µL of undiluted Sample Purification Beads (AMPure, Illumina) were used. Furthermore, for final library enrichment, a total of 9 amplification cycles were performed. Size distribution of the enriched libraries was determined on the Fragment Analyzer system (Advanced Analytical Technologies, Oak Tree, Ankeny, IA, USA) using the DNF-930 dsDNA Reagent Kit (75–20,000 bp) and concentration was determined by fluorometric quantification using QuBit 3.0 (ThermoFisher Scientific, Waltham, MA, USA). Pooled libraries were clustered with a concentration of 12 pM on a Standard V2 Flow Cell and sequenced in two runs using the MiSeq Reagent Kit v2 (500 cycles) and in one run on an Illumina HiSeq 2500.

### 4.5. Bioinformatic Analysis Pipeline for NGS mtDNA Sequencing Data

The current analysis was conducted based on the FASTQ files, subsequently aligned/mapped to the rCRS reference with bwa-mem [59] and converted with samtools [60] to BAM files. Quality controls were performed on the FASTQ files with FastQC and QualiMap was employed on the BAM files. The respective reports were aggregated with MultiQC into a merged quality report. The subsequent processing of the BAM files with the mutserve v1.3.0 (https://github.com/seppinho/mutserve) resulted in the reporting on heteroplasmy frequencies and variants. QC reports on the overall data quality were generated in R. Additionally, potential within-sample contamination caused by mix-ups was analyzed with haplocheck (manuscript submitted for publication [28], https://mitoverse.i-med.ac.at/). Further, by analyzing the data on the mtDNA-Server (http://mtdna-server.uibk.ac.at), the web-service automatically annotates known sites for which the presence of NUMTs (nuclear inserts of mitochondrial genes) was previously described [61].

### 4.6. Statistical Analysis

We compared heteroplasmic levels of tumor and benign tissues using the Wilcoxon test and Fisher’s exact test.

Individuals were divided according to smoking behavior in smokers (current and former smokers) and no smokers (those who have never smoked).

Survival analyses were performed for carriers and non-carriers of mtDNA mutations in the coding region (mut_coding). Overall survival was estimated by Kaplan–Meier curves and the log-rank test. Cox’s proportional hazards models were fitted without adjustment and with adjustment for smoking status, since it is a strong risk factor for OSCC. A further sensitivity analysis was adjusted for N1 based on the results of the univariate analyses. Further adjustment was restricted by the small number of events.

Statistical analysis was performed using R 3.3.2 (Vienna, Austria, http://www.R-project.org), by generating *markdown* reports, directly based on the mutserve output and clinical parameters. The packages *survival* and *survminer* were used for survival analysis, by computing both, Kaplan–Meier curves, as well as the Cox-regression analysis. Statistical significance was set at alpha = 0.05.

## 5. Conclusions

We conclude that heteroplasmic variants in mitochondrial coding genes are predominant in OSCC tumors compared to the benign counterparts. Particularly higher heteroplasmic mtDNA variants are enriched in the tumors. Potentially deleterious high heteroplasmic level mutations in protein-coding regions are significantly associated with shorter patient survival. In OSCC cancer patients, haplogroup H was more frequent than in the general population. This calls for larger studies to verify the impact of haplogroups on OSCC risk.

## Figures and Tables

**Table 1:** Patient characteristics. Females represented 33.3% of the study group. A total of 81% of the material was collected from primary tumors; 38.9% of the individuals were active smokers; 38.9% of patients had a T grade of 4, 38.9% of 2, and 22.2% had a T grade of 1. 33.3% of all patients had a lymph node involvement; 5.4% presented distant metastases.

Sample ID	Age at Diagnosis	Gender	Material Used	Smoking Status	Time to Death after Diagnosis (Months)	Follow Up Time (Months)	T	N	M	R	Grading	Coding-Region Mutations	Haplogroup
MKG1	32	F	Primary tumor	no-smoker	-	128	2	0	0	0	3	yes	H1
MKG2	71	F	Primary tumor	no-smoker	-	118	1	0	0	0	2	no	U5
MKG4	70	M	Primary tumor	active smoker	64	64	4a	1	0	1	3	no	H16
MKG5	73	M	Primary tumor	former smoker	107	107	4a	0	NA	1	2	no	K1
MKG6	58	M	Primary tumor	active smoker	27	27	4a	1	1	0	2	yes	H1
MKG7	59	M	Primary tumor	former smoker	-	119	4a	0	0	0	2	no	H27
MKG8	51	F	Recurrence	active smoker	38	38	4	0	0	1	2	yes	H11
MKG9	62	F	Primary tumor	no-smoker	-	114	1	0	0	0	1	no	J1
MKG10	60	M	Recurrence	former smoker	47	47	4a	0	0	0	2	no	H13
MKG11	53	M	Primary tumor	former smoker	-	116	4	0	0	0	3	no	J1
MKG12	50	M	Primary tumor	no-smoker	-	54	2	1	0	0	2	no	H11
MKG13	64	F	Recurrence	no-smoker	83	83	4a	0	0	0	2	no	T1
MKG14	63	M	Primary tumor	active smoker	-	111	4a	0	0	1	NA	no	H1
MKG15	64	M	Primary tumor	active smoker	6	6	1	2a	0	0	3	yes	H3
MKG16	49	M	Primary tumor	former smoker	-	98	1	0	0	0	3	no	U4
MKG17	68	F	Primary tumor	no-smoker	-	43	2	0	0	0	2	no	H5
MKG18	68	M	Primary tumor	former smoker	-	97	2	1	0	0	2	no	T2
MKG19	37	M	Recurrence	no-smoker	36	36	4a	0	0	0	3	no	H1
MKG20	57	M	Primary tumor	active smoker	7	7	4a	1	0	1	3	yes	H
MKG21	75	F	Primary tumor	no-smoker	23	23	2	0	0	0	2	no	U5
MKG22	67	M	Primary tumor	former smoker	-	103	2	0	0	0	2	yes	U5
MKG23	56	M	Primary tumor	former smoker	42	42	2	1	0	0	3	no	W1
MKG24	45	M	Primary tumor	active smoker	13	13	4a	0	0	0	2	no	H5
MKG25	66	F	Primary tumor	former smoker	1	1	2	0	0	0	2	yes	H3
MKG26	62	M	Primary tumor	former smoker	-	45	2	2b	0	0	2	no	H
MKG27	59	F	Primary tumor	active smoker	15	15	2	2b	0	0	2	no	H3
MKG28	73	M	Recurrence	active smoker	27	27	2	0	0	0	2	no	H1
KT000	60	M	Recurrence	former smoker	-	139	1	0	0	0	2	yes	H65
KT001	54	F	Primary tumor	active smoker	1	1	4b	2c	1	NA	2	yes	J1
KT003	72	M	Primary tumor	active smoker	-	70	2	2b	0	1	2	no	K2
KT007	45	M	Primary tumor	active smoker	16	16	2	0	0	0	2	no	H3
KT008	83	F	Primary tumor	no-smoker	-	65	1	0	0	0	0	no	U2
KT012	50	M	Primary tumor	active smoker	-	52	2	0	0	0	3	no	H3
KT013	73	M	Recurrence	no-smoker	-	104	1	0	0	0	2	no	H1
KT014	57	F	Primary tumor	active smoker	-	41	1	0	0	0	2	yes	K2
KT016	62	M	Primary tumor	no-smoker	-	39	4a	1	0	0	3	yes	H10

## Supplementary Materials

The following are available online at https://www.mdpi.com/2072-6694/12/7/1933/s1. Table S1: List of all the variants identified in the 74 tissue samples with their level of heteroplasmy, coverage, sequencing run and locus (heteroplasmy threshold ≥ 0.01). Table S2: Mutations with at least 20% (∆HF% > 20) absolute percentage point difference in heteroplasmy between tumor and paired benign samples. Table S3: Univariate analyses of cox proportional-hazard models for mut_coding and other potential risk factors, Figure S1: Kaplan–Meier survival curve for OSCC patients belonging to Haplogroup H compared to patients of all the other haplogroups, Figure S2: Age distributions among different macro Haplogroup clusters, H including all haplogroups under H (*n* = 22), JT (*n* = 5) combines all sequences under haplogroups J and T, Uk (*n* = 8) includes all samples under haplogroups U and K, as well as haplogroup W (*n* = 1), which comprised 1 sample only, Figure S3: ∆HF% > 20 variants (delta_mutations) with a trend towards more delta_mutations as age increases, Figure S4: Samples with a tumor grading of 2 had a significantly higher age (median 62 years) compared to patients with a tumor grading of 3 (median 54.5 years) at time of first diagnosis.

## References

[1] Sun J. (2019). RASSF-1A modulates proliferation-mediated oral squamous cell carcinoma progression. Cancer Cell Int..

[2] Hema K., Smitha T., Sheethal H., Mirnalini S.A. (2017). Epigenetics in oral squamous cell carcinoma. J. Oral Maxillofac. Pathol..

[3] Ishida K., Tomita H., Nakashima T., Hirata A., Tanaka T., Shibata T., Hara A. (2017). Current mouse models of oral squamous cell carcinoma: Genetic and chemically induced models. Oral Oncol..

[4] Máximo V., Soares P., Lima J., Cameselle-Teijeiro J., Sobrinho-Simões M. (2002). Mitochondrial DNA somatic mutations (point mutations and large deletions) and mitochondrial DNA variants in human thyroid pathology: A study with emphasis on Hürthle cell tumors. Am. J. Pathol..

[5] Loogväli E.L., Kivisild T., Margus T., Villems R. (2009). Explaining the imperfection of the molecular clock of hominid mitochondria. PLoS ONE.

[6] Kivisild T., Shen P., Wall D.P., Do B., Sung R., Davis K., Passarino G., Underhill P.A., Scharfe C., Torroni A. (2006). The role of selection in the evolution of human mitochondrial genomes. Genetics.

[7] Kivisild T., Reidla M., Metspalu E., Rosa A., Brehm A., Pennarun E., Parik J., Geberhiwot T., Usanga E., Villems R. (2004). Ethiopian mitochondrial DNA heritage: Tracking gene flow across and around the gate of tears. Am. J. Hum. Genet..

[8] Behar D.M., Villems R., Soodyall H., Blue-Smith J., Pereira L., Metspalu E., Scozzari R., Makkan H., Tzur S., Comas D. (2008). The Dawn of Human Matrilineal Diversity. Am. J. Hum. Genet..

[9] Salas A., Yao Y.G., Macaulay V., Vega A., Carracedo Á., Bandelt H.J. (2005). A critical reassessment of the role of mitochondria in tumorigenesis. PLoS Med..

[10] Bussard K.M., Siracusa L.D. (2017). Understanding Mitochondrial Polymorphisms in Cancer. Cancer Res..

[11] Kloss-Brandstätter A., Schäfer G., Erhart G., Hüttenhofer A., Coassin S., Seifarth C., Summerer M., Bektic J., Klocker H., Kronenberg F. (2010). Somatic mutations throughout the entire mitochondrial genome are associated with elevated PSA levels in prostate cancer patients. Am. J. Hum. Genet..

[12] Fendt L., Niederstätter H., Huber G., Zelger B., Dünser M., Seifarth C., Röck A., Schäfer G., Klocker H., Parson W. (2011). Accumulation of mutations over the entire mitochondrial genome of breast cancer cells obtained by tissue microdissection. Breast Cancer Res. Treat..

[13] Schöpf B., Weissensteiner H., Schäfer G., Fazzini F., Charoentong P., Naschberger A., Rupp B., Fendt L., Bukur V., Giese I. (2020). OXPHOS remodeling in high-grade prostate cancer involves mtDNA mutations and increased succinate oxidation. Nat. Commun..

[14] Kloss-Brandstätter A., Weissensteiner H., Erhart G., Schäfer G., Forer L., Schönherr S., Pacher D., Seifarth C., Stöckl A., Fendt L. (2015). Validation of Next-Generation Sequencing of Entire Mitochondrial Genomes and the Diversity of Mitochondrial DNA Mutations in Oral Squamous Cell Carcinoma. PLoS ONE.

[15] Challen C., Brown H., Cai C., Betts G., Paterson I., Sloan P., West C., Birch-Machin M., Robinson M. (2011). Mitochondrial DNA mutations in head and neck cancer are infrequent and lack prognostic utility. Br. J. Cancer.

[16] Uzawa K., Baba T., Uchida F., Yamatoji M., Kasamatsu A., Sakamoto Y., Ogawara K., Shiiba M., Bukawa H., Tanzawa H. (2012). Circulating tumor-derived mutant mitochondrial DNA: A predictive biomarker of clinical prognosis in human squamous cell carcinoma. Oncotarget.

[17] Lai C.H., Huang S.F., Liao C.T., Chen I.H., Wang H.M., Hsieh L.L. (2013). Clinical Significance in Oral Cavity Squamous Cell Carcinoma of Pathogenic Somatic Mitochondrial Mutations. PLoS ONE.

[18] Mondal R., Ghosh S.K. (2013). Accumulation of mutations over the complete mitochondrial genome in tobacco-related oral cancer from northeast India. Mitochondrial DNA.

[19] Gissi D.B., Tarsitano A., Leonardi E., Gabusi A., Neri F., Marchetti C., Montebugnoli L., Foschini M.P., Morandi L. (2017). Clonal analysis as a prognostic factor in multiple oral squamous cell carcinoma. Oral Oncol..

[20] Morandi L., Tarsitano A., Gissi D., Leonardi E., Balbi T., Marchetti C., Montebugnoli L., Foschini M.P. (2015). Clonality analysis in primary oral squamous cell carcinoma and related lymph-node metastasis revealed by TP53 and mitochondrial DNA next generation sequencing analysis. J. Cranio-Maxillofac. Surg..

[21] Yuan R.T., Sun Y., Bu L.X., Jia M.Y. (2015). Gene mutations in the D-loop region of mitochondrial DNA in oral squamous cell carcinoma. Mol. Med. Rep..

[22] Lin J.C., Wang C.C., Jiang R.S., Wang W.Y., Liu S.A. (2015). Impact of somatic mutations in the D-Loop of mitochondrial DNA on the survival of oral squamous cell carcinoma patients. PLoS ONE.

[23] Palodhi A., Ghosh S., Biswas N.K., Basu A., Majumder P.P., Maitra A. (2019). Profiling of genomic alterations of mitochondrial DNA in gingivobuccal oral squamous cell carcinoma: Implications for disease progress. Mitochondrion.

[24] Schubert A.D., Channah Broner E., Agrawal N., London N., Pearson A., Gupta A., Wali N., Seiwert T.Y., Wheelan S., Lingen M. (2020). Somatic mitochondrial mutation discovery using ultra-deep sequencing of the mitochondrial genome reveals spatial tumor heterogeneity in head and neck squamous cell carcinoma. Cancer Lett..

[25] Bandelt H.J., Salas A. (2009). Contamination and sample mix-up can best explain some patterns of mtDNA instabilities in buccal cells and oral squamous cell carcinoma. BMC Cancer.

[26] Weissensteiner H., Forer L., Fuchsberger C., Schöpf B., Kloss-Brandstätter A., Specht G., Kronenberg F., Schönherr S. (2016). mtDNA-Server: Next-generation sequencing data analysis of human mitochondrial DNA in the cloud. Nucleic Acids Res..

[27] Weissensteiner H., Pacher D., Kloss-Brandstätter A., Forer L., Specht G., Bandelt H.-J., Kronenberg F., Salas A., Schönherr S. (2016). HaploGrep 2: Mitochondrial haplogroup classification in the era of high-throughput sequencing. Nucleic Acids Res..

[28] Weissensteiner H., Forer L., Fendt L., Kheirkhah A., Salas A., Kronenberg F., Schoenherr S. (2020). Haplocheck: Phylogeny-based Contamination Detection in Mitochondrial and Whole-Genome Sequencing Studies. bioRxiv.

[29] Van Oven M. (2015). PhyloTree Build 17: Growing the human mitochondrial DNA tree. Forensic Sci. Int. Genet. Suppl. Ser..

[30] Van Oven M., Kayser M., van Oven M., Kayser M. (2009). Updated comprehensive phylogenetic tree of global human mitochondrial DNA variation. Hum. Mutat..

[31] Wei W., Pagnamenta A.T., Gleadall N., Sanchis-Juan A., Stephens J., Broxholme J., Tuna S., Odhams C.A., Fratter C., Turro E. (2020). Nuclear-mitochondrial DNA segments resemble paternally inherited mitochondrial DNA in humans. Nat. Commun..

[32] Salas A., Schönherr S., Bandelt H.-J., Gómez-Carballa A., Weissensteiner H. (2020). Extraordinary claims require extraordinary evidence in asserted mtDNA biparental inheritance. Forensic Sci. Int. Genet..

[33] Balciuniene J., Balciunas D. (2019). A Nuclear mtDNA Concatemer (Mega-NUMT) Could Mimic Paternal Inheritance of Mitochondrial Genome. Front. Genet..

[34] Wallace D.C., Chalkia D. (2013). Mitochondrial DNA genetics and the heteroplasmy conundrum in evolution and disease. Cold Spring Harb. Perspect. Biol..

[35] Hopkins J., Sabelnykova V., Weischenfeldt J., Simon R., Aguiar J., Alkallas R., Heisler L., Zhang J., Watson J., Chua M. (2017). Mitochondrial mutations drive prostate cancer aggression. Nat. Commun..

[36] Pereira L., Soares P., Radivojac P., Li B., Samuels D.C. (2011). Comparing Phylogeny and the Predicted Pathogenicity of Protein Variations Reveals Equal Purifying Selection across the Global Human mtDNA Diversity. Am. J. Hum. Genet..

[37] Vigneswaran N., Williams M.D. (2014). Epidemiologic Trends in Head and Neck Cancer and Aids in Diagnosis. Oral Maxillofac. Surg. Clin. North Am..

[38] Ebner S., Mangge H., Langhof H., Halle M., Siegrist M., Aigner E., Paulmichl K., Paulweber B., Datz C., Sperl W. (2015). Mitochondrial haplogroup T is associated with obesity in Austrian juveniles and adults. PLoS ONE.

[39] Ye K., Lu J., Ma F., Keinan A., Gu Z. (2014). Extensive pathogenicity of mitochondrial heteroplasmy in healthy human individuals. Proc. Natl. Acad. Sci. USA.

[40] Skonieczna K., Malyarchuk B., Jawień A., Marszałek A., Banaszkiewicz Z., Jarmocik P., Grzybowski T. (2018). Mitogenomic differences between the normal and tumor cells of colorectal cancer patients. Hum. Mutat..

[41] Carinci F., Pelucchi S., Farina A., De Franciscis G., Calearo C. (1998). Extension as a prognostic factor in oropharyngeal cancer: Largest mucosal dimension compared with number of (sub)sites involved. Br. J. Oral Maxillofac. Surg..

[42] McMahon J., O’Brien C.J., Pathak I., Hamill R., McNeil E., Hammersley N., Gardiner S., Junor E. (2003). Influence of condition of surgical margins on local recurrence and disease-specific survival in oral and oropharyngeal cancer. Br. J. Oral Maxillofac. Surg..

[43] Byers R.M., El-Naggar A.K., Lee Y.-Y., Rao B., Fornage B., Terry N.H.A., Sample D., Hankins P., Smith T.L., Wolf P.J. (1998). Can we detect or predict the presence of occult nodal metastases in patients with squamous carcinoma of the oral tongue?. Head Neck.

[44] Woolgar J.A., Rogers S.N., Lowe D., Brown J.S., Vaughan E.D. (2003). Cervical lymph node metastasis in oral cancer: The importance of even microscopic extracapsular spread. Oral Oncol..

[45] Suoglu Y., Erdamar B., Karatay M.C., Katircioglu O.S., Sunay T. (2002). Extracapsular Spread in Ipsilateral Neck and Contralateral Neck Metastases in Laryngeal Cancer. Ann. Otol. Rhinol. Laryngol..

[46] Enepekides D.J., Sultanem K., Nguyen C., Shenouda G., Black M.J., Rochon L. (1999). Occult Cervical Metastases: Immunoperoxidase Analysis of the Pathologically Negative Neck. Otolaryngol. Neck Surg..

[47] Lin H.-S., Siddiq F., Talwar H.S., Chen W., Voichita C., Draghici S., Jeyapalan G., Chatterjee M., Fribley A., Yoo G.H. (2014). Serum prognostic biomarkers in head and neck cancer patients. Laryngoscope.

[48] Lin H.-S., Talwar H.S., Tarca A.L., Ionan A., Chatterjee M., Ye B., Wojciechowski J., Mohapatra S., Basson M.D., Yoo G.H. (2007). Autoantibody Approach for Serum-Based Detection of Head and Neck Cancer. Cancer Epidemiol. Biomark. Prev..

[49] Gottschlich S., Maune S., Maass J.D., Görögh T., Hoffmann M., Hoffmann-Fazel A., Meyer J., Werner J.A., Rudert H. (2000). Serum p53 Autoantibodies in the Follow-Up of Head and Neck Cancer Patients. Oncology.

[50] Dasgupta S., Koch R., Westra W.H., Califano J.A., Ha P.K., Sidransky D., Koch W.M. (2010). Mitochondrial DNA Mutation in Normal Margins and Tumors of Recurrent Head and Neck Squamous Cell Carcinoma Patients. Cancer Prev. Res..

[51] Kumar M., Srivastava S., Singh S.A., Das A.K., Das G.C., Dhar B., Ghosh S.K., Mondal R. (2017). Cell-free mitochondrial DNA copy number variation in head and neck squamous cell carcinoma: A study of non-invasive biomarker from Northeast India. Tumor Biol..

[52] Cicchillitti L., Corrado G., De Angeli M., Mancini E., Baiocco E., Patrizi L., Zampa A., Merola R., Martayan A., Conti L. (2017). Circulating cell-free DNA content as blood based biomarker in endometrial cancer. Oncotarget.

[53] Creed J., Klotz L., Harbottle A., Maggrah A., Reguly B., George A., Gnanapragasm V. (2017). A single mitochondrial DNA deletion accurately detects significant prostate cancer in men in the PSA ‘grey zone’. World J. Urol..

[54] Kirches E. (2017). MtDNA As a Cancer Marker: A Finally Closed Chapter?. Curr. Genom..

[55] Lu H., Giordano F., Ning Z. (2016). Oxford Nanopore MinION Sequencing and Genome Assembly. Genom. Proteom. Bioinform..

[56] Li M., Schröder R., Ni S., Madea B., Stoneking M. (2015). Extensive tissue-related and allele-related mtDNA heteroplasmy suggests positive selection for somatic mutations. Proc. Natl. Acad. Sci. USA.

[57] Aoki K., Tanaka H., Kawahara T. (2018). Multiplexed Microsphere Suspension-Array Assay for Urine Mitochondrial DNA Typing by C-Stretch Length in Hypervariable Regions. J. Clin. Med. Res..

[58] Fendt L., Zimmermann B., Daniaux M., Parson W. (2009). Sequencing strategy for the whole mitochondrial genome resulting in high quality sequences. BMC Genom..

[59] Li H., Durbin R. (2010). Fast and accurate long-read alignment with Burrows-Wheeler transform. Bioinformatics.

[60] Li H., Handsaker B., Wysoker A., Fennell T., Ruan J., Homer N., Marth G., Abecasis G., Durbin R. (2009). The Sequence Alignment/Map format and SAMtools. Bioinformatics.

[61] Dayama G., Emery S.B., Kidd J.M., Mills R.E. (2014). The genomic landscape of polymorphic human nuclear mitochondrial insertions. Nucleic Acids Res..

