# Supplementary material for: Profiling of Mitochondrial DNA Heteroplasmy in a Prospective Oral Squamous Cell Carcinoma Study
Source: Cancers (Basel). 2020 Jul 17;12(7):1933. doi: 10.3390/cancers12071933 (PMC7409097; doi:10.3390/cancers12071933)
Supplement: Supplementary file 1 [file cancers-12-01933-s001.zip › Supplemental Material/Supplemental-Material.docx]

**Table S2.** Mutations with at least 20% (∆HF>20%) absolute percentage point difference in heteroplasmy between tumor and paired benign samples.

| **Mutation** | **Sample ID** | **Variant** | **Variant Level BE** | **Variant level CA** | **Substitution** | **Locus** | **Aminoacid** | **New Aminoacid** | **Pathogenicity Score (MutPred)** |
| --- | --- | --- | --- | --- | --- | --- | --- | --- | --- |
| 64T | KT003 | T | 0.00 | 0.59 | transition | MT-DLOOP2 | - | - | - |
| 72C | KT016 | C | 0.35 | 0.00 | transition | MT-DLOOP2 | - | - | - |
| 72C | KT000 | C | 0.47 | 0.03 | transition | MT-DLOOP2 | - | - | - |
| 72C | MKG4 | C | 0.60 | 0.00 | transition | MT-DLOOP2 | - | - | - |
| 72C | MKG20 | C | 0.27 | 0.00 | transition | MT-DLOOP2 | - | - | - |
| 146C | MKG11 | C | 0.99 | 0.46 | transition | MT-DLOOP2 | - | - | - |
| 189G | KT016 | G | 0.00 | 0.87 | transition | MT-DLOOP2 | - | - | - |
| 456T | MKG11 | T | 0.01 | 0.61 | transition | MT-DLOOP2 | - | - | - |
| 1793A | MKG22 | A | 0.00 | 0.52 | transition | MT-RNR2 | - | - | - |
| 2164T | MKG11 | T | 0.00 | 0.57 | transition | MT-RNR2 | - | - | - |
| 2916A | MKG20 | A | 0.00 | 0.21 | transition | MT-RNR2 | - | - | - |
| 3180G | KT008 | G | 0.00 | 0.76 | transition | MT-RNR2 | - | - | - |
| 3483A | KT000 | A | 0.00 | 0.48 | transition | MT-ND1 | - | - | - |
| 3526A | MKG6 | A | 0.00 | 0.60 | transition | MT-ND1 | A | T | 0.618 |
| 4196T | KT014 | T | 0.00 | 0.56 | transition | MT-ND1 | T | I | 0.716 |
| 4476T | MKG22 | T | 0.00 | 0.56 | transition | MT-ND2 | P | S | 0.533 |
| 4835T | KT001 | T | 0.00 | 0.85 | transition | MT-ND2 | - | - | - |
| 5297T | MKG15 | T | 0.00 | 0.46 | transition | MT-ND2 | - | - | - |
| 6978A | KT016 | A | 0.00 | 0.89 | transition | MT-CO1 | A | T | 0.7 |
| 8865A | MKG6 | A | 0.28 | 0.66 | transition | MT-ATP6 | - | - | - |
| 9868A | KT014 | A | 0.00 | 0.58 | transition | MT-CO3 | R | H | 0.818 |
| 11682A | MKG25 | A | 0.00 | 0.74 | transition | MT-ND4 | S | N | 0.703 |
| 12736A | MKG20 | A | 0.00 | 0.25 | transition | MT-ND5 | A | T | 0.686 |
| 12868A | MKG15 | A | 0.00 | 0.38 | transition | MT-ND5 | G | S | 0.692 |
| 15355A | MKG8 | A | 0.53 | 0.11 | transition | MT-CYB | - | - | - |
| 15553A | MKG1 | A | 0.00 | 0.33 | transition | MT-CYB | - | - | - |
| 15969T | MKG14 | T | 0.00 | 0.82 | transition | MT-TP | - | - | - |
| 16147T | MKG21 | T | 0.00 | 0.40 | transition | MT-DLOOP1 | - | - | - |
| 16148T | MKG22 | T | 0.31 | 0.06 | transition | MT-DLOOP1 | - | - | - |
| 16183C | KT008 | C | 0.66 | 0.00 | transversion | MT-DLOOP1 | - | - | - |
| 16261T | MKG5 | T | 0.01 | 0.87 | transition | MT-DLOOP1 | - | - | - |
| 16278T | MKG4 | T | 0.00 | 0.59 | transition | MT-DLOOP1 | - | - | - |
| 16465T | MKG21 | T | 0.90 | 0.42 | transition | MT-DLOOP1 | - | - | - |
| 16519C | KT008 | C | 1.00 | 0.23 | transition | MT-DLOOP1 | - | - | - |

**Table S3.** Univariate analyses of cox proportional-hazard models for mut_coding and other potential risk factors.

| **Parameter** | **beta** | **HR (95% CI for HR)** | **p-value** |
| --- | --- | --- | --- |
| **∆HF>20% All [yes \| no]** | 0.98 | 2.7 (0.83-8.5) | 0.099 |
| **∆HF>20% Coding [yes \| no]** | 0.59 | 1.8 (0.54-6) | 0.33 |
| **Age (continuous)** | -0.0057 | 0.99 (0.95-1) | 0.82 |
| **Age60 [<60 \| >= 60]** | -0.59 | 0.55 (0.17-1.8) | 0.32 |
| **Gender [f \| m]** | -0.059 | 0.94 (0.28-3.1) | 0.92 |
| **Haplogroup [other \| H]** | -0.77 | 0.46 (0.14-1.6) | 0.22 |
| **Smokers [yes \| no]** | 1.1 | 3 (0.64-14) | 0.16 |
| **Staging T [4, 4a, 4b \| 0,1,2]** | 0.65 | 1.9 (0.61-6) | 0.26 |
| **Staging N [1, 2 \| 0]** | 1.1 | 3.1 (0.9-11) | 0.072 |
| **Grading (continuous)** | 0.32 | 1.4 (0.58-3.3) | 0.47 |


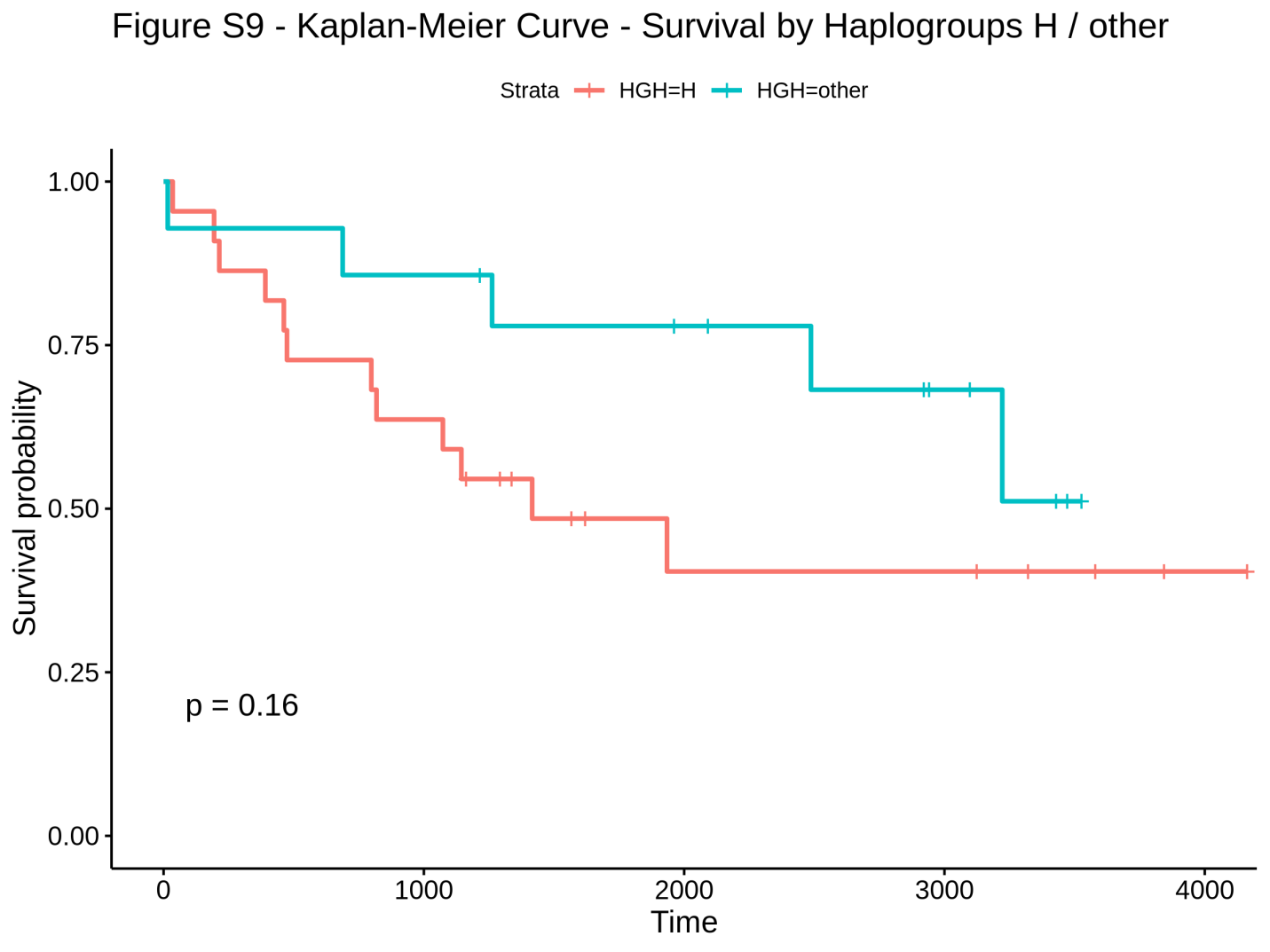


**Figure S1.** Kaplan Meier survival curve for OSCC patients belonging to Haplogroup H compared to patients of all the others haplogroups.


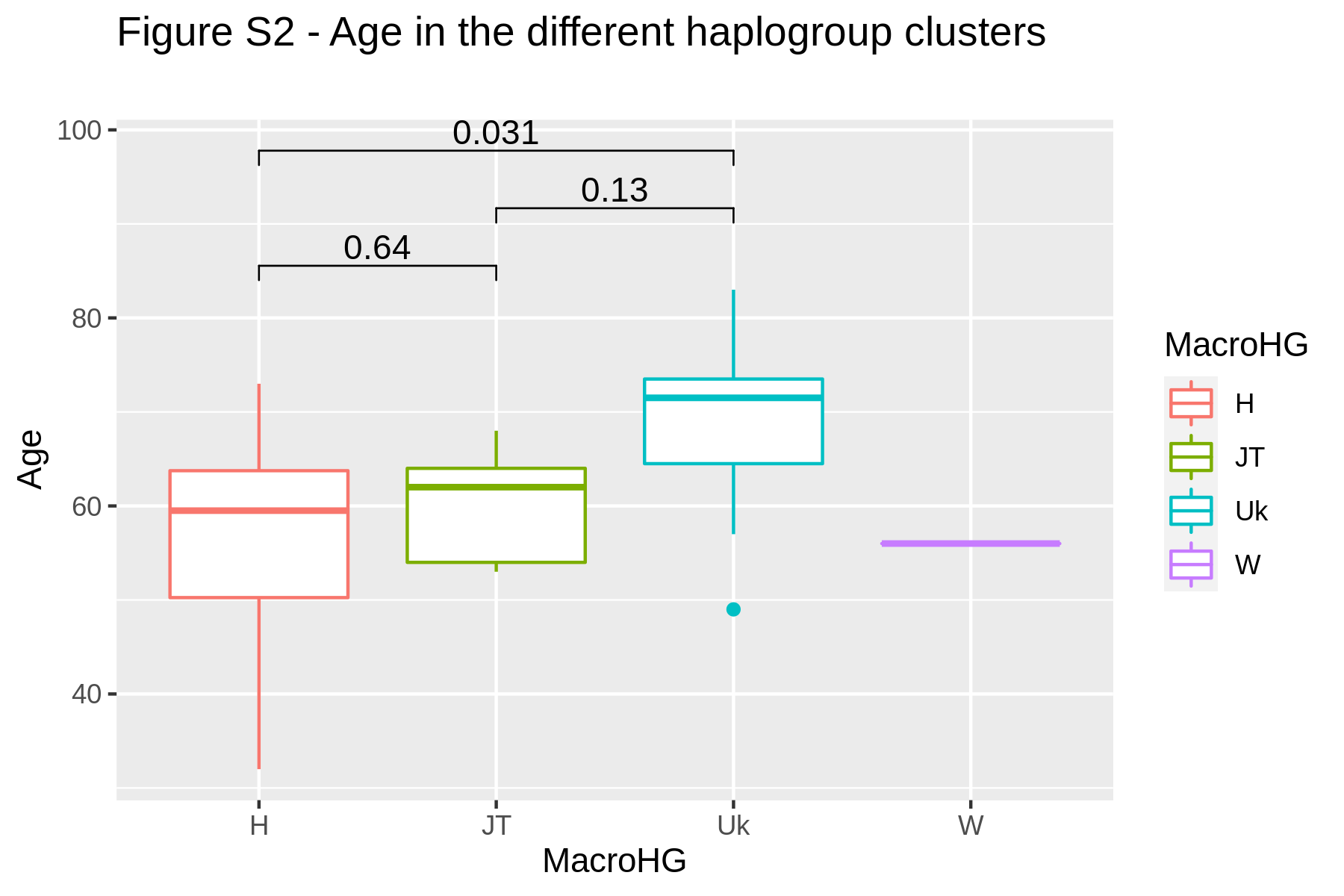


**Figure S2.** Age distributions among different macro Haplogroup clusters, H including all haplogroups under H (n=22), JT (n=5) combines all sequences under haplogroups J and T, Uk (n=8) includes all samples under haplogroups U and K, as well as haplogroup W (n=1), which comprised 1 sample only.


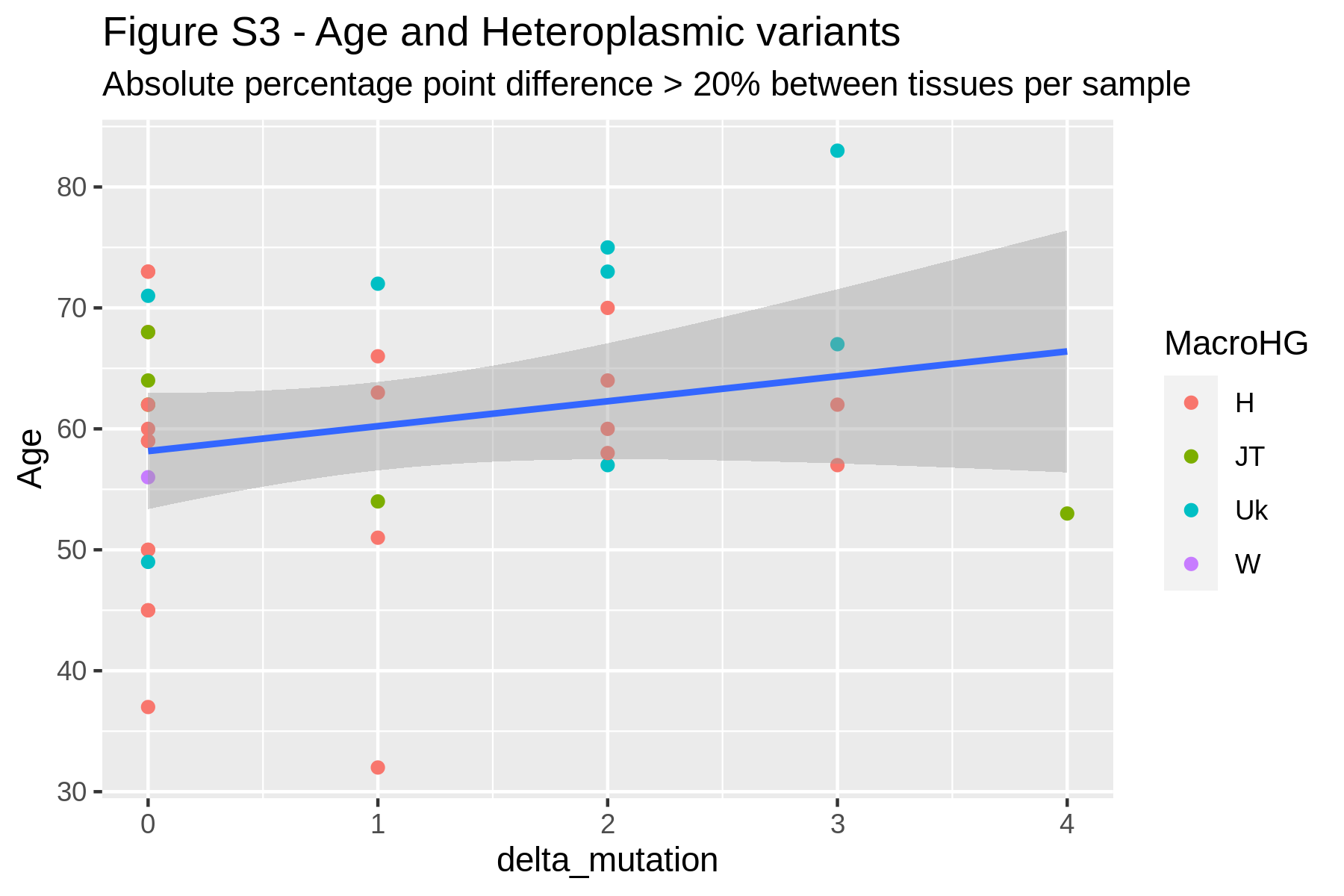


**Figure S3.** Variants with absolute percentage point difference of >20% (delta_mutations) between tumor and benign tissues, with a trend towards increased age with more delta_mutations.


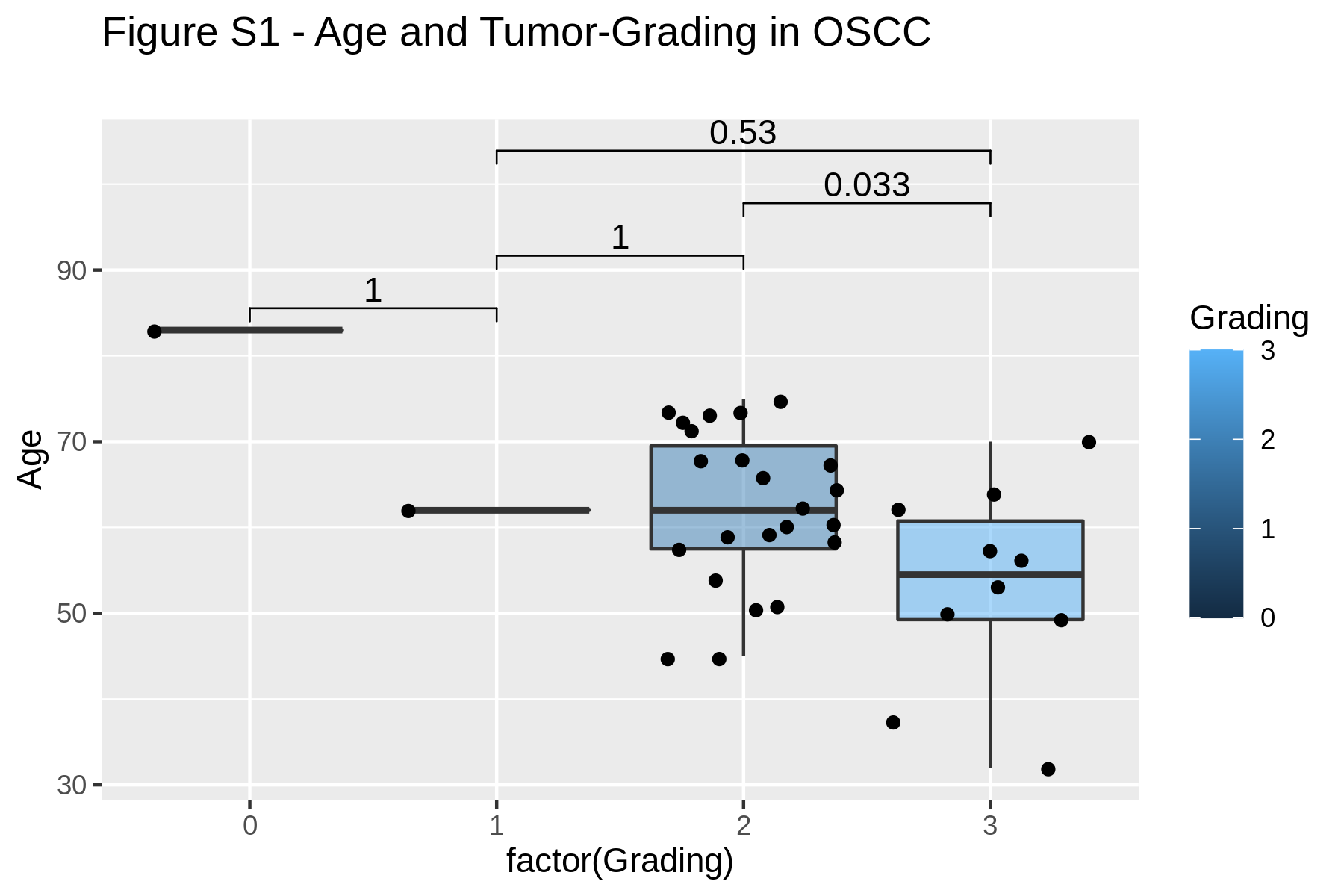


**Figure S4.** Samples with tumor grading 2 had a significantly higher age (median 62 years) compared to patients with tumor grading 3 (median 54.5 years) at time of first diagnose.
